# Thinking beyond the virus: perspective of patients on the quality of hospital care before and during the COVID-19 pandemic

**DOI:** 10.3389/fpubh.2023.1152054

**Published:** 2023-09-08

**Authors:** Eliza Lai-yi Wong, Kailu Wang, Annie Wai-ling Cheung, Chris Graham, Eng-kiong Yeoh

**Affiliations:** ^1^JC School of Public Health and Primary Care, Faculty of Medicine, The Chinese University of Hong Kong, Hong Kong SAR, Hong Kong SAR, China; ^2^Centre for Health Systems and Policy Research, JC School of Public Health and Primary Care, Faculty of Medicine, The Chinese University of Hong Kong, Hong Kong SAR, Hong Kong SAR, China; ^3^Picker Institute Europe, Oxford, United Kingdom

**Keywords:** COVID-19, patient experience, hospitalization, person-centred care, nurse–patient relationship

## Abstract

**Objectives:**

The COVID-19 pandemic has a huge impact on the healthcare system and affects the normal delivery of routine healthcare services to hospitalized patients. This study aimed to examine the differences in patient experience of hospital service before and during COVID-19 among the discharged adult population.

**Methods:**

A territory-wide patient experience survey was conducted before and during COVID-19 (between October 2019 and April 2020) among patients discharged from the main acute and rehabilitation public hospitals in Hong Kong. A hierarchical ordinal logistic model was employed to examine the difference in multiple dimensions of patient experience, with adjustments of covariates.

**Results:**

In total, 9,800 participants were recruited. During the pandemic, there was a marginally significant increase in overall care rating (AOR: 1.12, 95% CI: 0.99–1.27), and an improvement in the timeliness of admission. However, significant reductions in patients’ confidence in nurses were observed. Communication of information regarding medication side effects reduced significantly (AOR: 0.72, 95% CI: 0.64–0.82).

**Conclusion:**

The patients hospitalized during the pandemic reported worse responsiveness in communication in their patient journey than those admitted before the pandemic. These findings will help develop appropriate strategies to address patients’ concerns in the new normal.

## 1. Introduction

The COVID-19 pandemic has continuously affected the delivery of healthcare services and the mode of hospital operation (1–3). It has caused a great impact on hospital capacity for isolation wards, intensive care units, emergency departments, and treatment of unstable patients (4, 5). A wide range of scheduled care services have been cancelled and the utilization of non-COVID-19 healthcare services has decreased dramatically (6, 7). Hospitals have also changed their operating procedures for infection control and increased capacity for hospital care, including the universal use of personal protective equipment (PPE) among staff, suspension of visiting, and reduction of elective surgery rates (8). In Hong Kong (HK), all public hospitals elevated the emergency response level from “serious” to “emergency” on 25 January 2020, 2 days after the identification of the first COVID-19 case locally, involving a compulsory mask-wearing requirement for all individuals in healthcare facilities, the suspension of visiting and volunteer services in the hospitals, and an adjustment of non-emergency services (9). Under the circumstances of different infection control measures, communication between healthcare staff and patients and their family members was also influenced by the reduction of face-to-face contact, with the suspension of visiting, increased workload, and increased stress levels among healthcare workers (10, 11), especially in the hospital setting with the extra need for PPE during the pandemic (12) which may reduce the communicate between the parties. Some of the studies found that some hospitalized patient groups such as older adults or persons with mental health care may have a poorer experience (13, 14).

In light of these impacts, there is a need to determine the change in patient experience before and during the COVID-19 outbreak. Patient experience is an important component of healthcare quality, as it is usually associated with better health outcomes (15). Better patient experience is related to clinical safety and effectiveness, as well as lower mortality and readmission rates (16–18). Good patient experience is often facilitated by sufficient information, effective doctor-patient communication, timely response to patients’ needs, and patient involvement in decision-making, which improve the adherence and timeliness of treatment, and reduce unnecessary clinical actions (18, 19). Patients’ self-report of their experience will provide insights for healthcare providers to identify the advantages and disadvantages in their daily service provision, and provide ways to address these issues. In addition, understanding patient reported experiences in hospital during the COVID-19 pandemic may provide valuable insights into patient needs and inform future policy for the next pandemic.

Few studies reported the impact on patient experience before and during the COVID-19 pandemic. A survey conducted among the UK National Health Service (NHS) patients revealed that patients reported a better experience regarding emotional support and overall care rating during the pandemic, compared to the 2018 and 2019 surveys (before the pandemic), and there were no differences regarding the information they obtained and their experience during hospital discharge (20). Another study in England also reported a positive experience during the pandemic (21). However, the territory-wide study exploring the impact of the infection control measures during the pandemic on the patient-reported experience and comparing with the pre-pandemic situation in the hospital setting among the Chinese population is scarce. There is a need to examine the differences due to the pandemic using multiple aspects of patient experience measurements. Considering the research gaps, this study aimed to examine the differences in patient experience before and during the COVID-19 pandemic with a well-validated instrument, and to investigate whether health-related and service usage factors that affected their experience. The measurement of patient experience should cover hospital admission, hospital staff, care and treatment, discharge, and overall rating. Without effective treatment, COVID-19 may extend over a long period. The findings from this study can be relevant for hospital administrators in identifying the weakness in hospital care during the pandemic and improving the quality of care during this period.

## 2. Methods

A cross-sectional survey using a structured questionnaire was conducted among patients who were discharged from any one of all major acute and rehabilitation hospitals (27 public hospitals) under Hospital Authority in Hong Kong through telephone interviews between 24 October 2019 and 9 April 2020.

### 2.1. Study sample and data collection

The target population was the patients who were (1) aged 18 years or above on the date of admission, (2) stayed at least one night in the hospital, (3) discharged from any one of the 27 hospitals from 11 October 2019 to 3 April 2020, and (4) contacted within 14 days after discharge. Patients admitted to the Department of Psychiatry, Obstetrics, Dentistry, Hospice, Infirmary, Pediatrics, and Intensive Care Services were excluded. The participants were selected randomly from the eligible target patients and approached through telephone calls for initial consent for the survey. In addition, a proportional stratified sampling method according to the reference on the proportions of discharge numbers in each hospital compared to the overall target of discharge population during the survey period was applied to ensure adequate representativeness. The participants were interviewed using a structured questionnaire via telephone, and formal verbal consent was obtained prior to the survey. A reasonable sample size of more than 9,500 responses was deemed acceptable for the telephone survey, and was estimated to achieve a precision level of plus/minus 4.0% points for the patient experience level of discharged patients in each hospital (assuming that 50% of respondents had a positive experience with the hospital service) at a 95% confidence level. For each potential participant, at least 5 telephone calls were to be made if the target person was unavailable.

### 2.2. Instruments and variables

The Hong Kong Inpatient Experience Questionnaire (HKIEQ), was developed and validated for measuring patient experience in hospital care in Hong Kong (22, 23). The questionnaire involved questions regarding experience under five dimensions of the patient journey: (1) admission to hospital, (2) hospital staff, (3) patient care and treatment, (4) discharge from hospital, and (5) overall impression. In the overall impression dimension, a five-point Likert-type scale was used to measure the care received from doctors, nurses, allied health professionals, healthcare assistants, and overall care (very poor to very good). In the other four dimensions, patient experience on inpatient admission timeliness, confidence in hospital staff, communication with hospital staff, and information sharing before discharge from the hospital were measured using a three-point response scale (1 = no, 2 = to some extent/sometimes, 3 = completely/definitely/always). Date of admission was used to generate the indicator for the COVID-19 period, where the patients admitted on or after 25 January 2020 were considered as the “during COVID-19” group and those admitted before 25 January 2020 were considered as the “normal period” group, as public hospitals announced the elevation of the emergency response level from “serious” to “emergency” after the first local case identified on 23 January 2020, which would last throughout the entire survey period until 2021 (9). Under the “emergency” response level, visiting arrangements and volunteer services were suspended in all public hospitals, and non-emergency services were reviewed and adjusted by each hospital independently for focusing resources to cope with the COVID-19 situation. In addition, information regarding the source of hospital admission (emergency room or planned/hospital transfer), length of stay (LOS, 0–3 days, 4–7 days, over 7 days), self-rated health (5-point scale, very poor to very good), and demographics of the patients were obtained for analysis.

### 2.3. Statistical analysis

Data analyses were performed using the R package V. 4.0.3 and Stata V. 15.0 software. Descriptive demographic information of the discharged patients and their responses to the questions under the five patient experience dimensions were reported. Chi-square and Fisher’s exact tests were used to analyze the difference in experience before and during the COVID-19 pandemic. Two-way ANOVA (with residual and wild bootstrap), with adjustments for age and sex, was applied for the average score of overall impression rating to explore how the mean score changed according to the LOS and experience before/during COVID-19. A hierarchical ordinal logistic model, with participants nested within different hospitals, was performed to examine the difference in patient experience before/during COVID-19, with adjustment for admission source, LOS, self-rated health, interaction between experience before/during COVID-19 and LOS, and interaction between experience before/during COVID-19 and admission source, as well as demographic factors. Statistical significance was set at *p* < 0.05.

### 2.4. Ethical issues/statement

Ethical approvals were obtained from all the respective Clinical Research Ethics Committees under Hospital Authority. Verbal consent was obtained telephonically by the research staff before commencement of the interview. All respondents were informed about the purpose and research procedures of the study, and their rights in the study. Participants were allowed to refuse to answer questions or withdraw from the study at any time point. All the information was anonymous and was treated with strict confidentiality.

## 3. Results

### 3.1. Sample characteristics

In total, 9,800 participants provided valid responses to the survey, with a response rate of 40% (Table 1). Among the total, 47.6% were females, and 18.0, 39.1, 29.3, and 13.6% were aged 18–40 years, 41–60 years, 61–70 years, and ≥71 years, respectively. Most of them were admitted to the hospital through the emergency department (60.8%), with an LOS <3 days (68.7%). Majority of the participants reported their health status as “fair” (50.3%) or “good” (39.5%), while fewer reported their health status as “very good” (2.5%), “poor” (6.9%) or “very poor” (0.9%). Comparing these characteristics before and during COVID-19, there were slightly more participants aged 18–40 years (15.8% before COVID-19 vs. 21.3% during COVID-19), males (51.0 vs. 54.3%), patients admitted from the emergency department (57.7 vs.65.5%), patients with a LOS <3 days (67.2 vs. 70.9%), and patients who perceived their health status as very good (1.7 vs.3.7%) and fair (48.6 vs. 52.7%) during the COVID-19 pandemic.

**Table 1:** Characteristics of the survey sample.

	Before COVID-19 pandemic	During COVID-19 pandemic	Total
	*N* (%)	*N* (%)	*N* (%)
Age			
18–40 years	926 (15.8)	840 (21.3)	1,766 (18.0)
41–60 years	2,310 (39.5)	1,517 (38.5)	3,827 (39.1)
61–70 years	1,788 (30.5)	1,082 (27.4)	2,870 (29.3)
71+ years	832 (14.2)	505 (12.8)	1,337 (13.6)
Sex			
Male	2,989 (51.0)	2,143 (54.3)	5,132 (52.4)
Female	2,867 (49.0)	1,801 (45.7)	4,668 (47.6)
Admission source			
Acute admission	3,376 (57.7)	2,585 (65.5)	5,961 (60.8)
Non-acute admission	2,480 (42.4)	1,359 (34.5)	3,839 (39.2)
Length of stay			
0–3 days	3,933 (67.2)	2,795 (70.9)	6,728 (68.7)
4–7 days	949 (16.2)	587 (14.9)	1,536 (15.7)
>7 days	974 (16.6)	562 (14.3)	1,536 (15.7)
Self-rated health			
Very good	102 (1.7)	145 (3.7)	247 (2.5)
Good	2,400 (41.0)	1,469 (37.3)	3,869 (39.5)
Fair	2,847 (48.6)	2,078 (52.7)	4,925 (50.3)
Poor	456 (7.8)	215 (5.5)	671 (6.9)
Very poor	50 (0.9)	35 (0.9)	85 (0.9)
Total	5,856 (100.0)	3,944 (100.0)	9,800 (100.0)

### 3.2. Hospital admission

**Figure 1:**
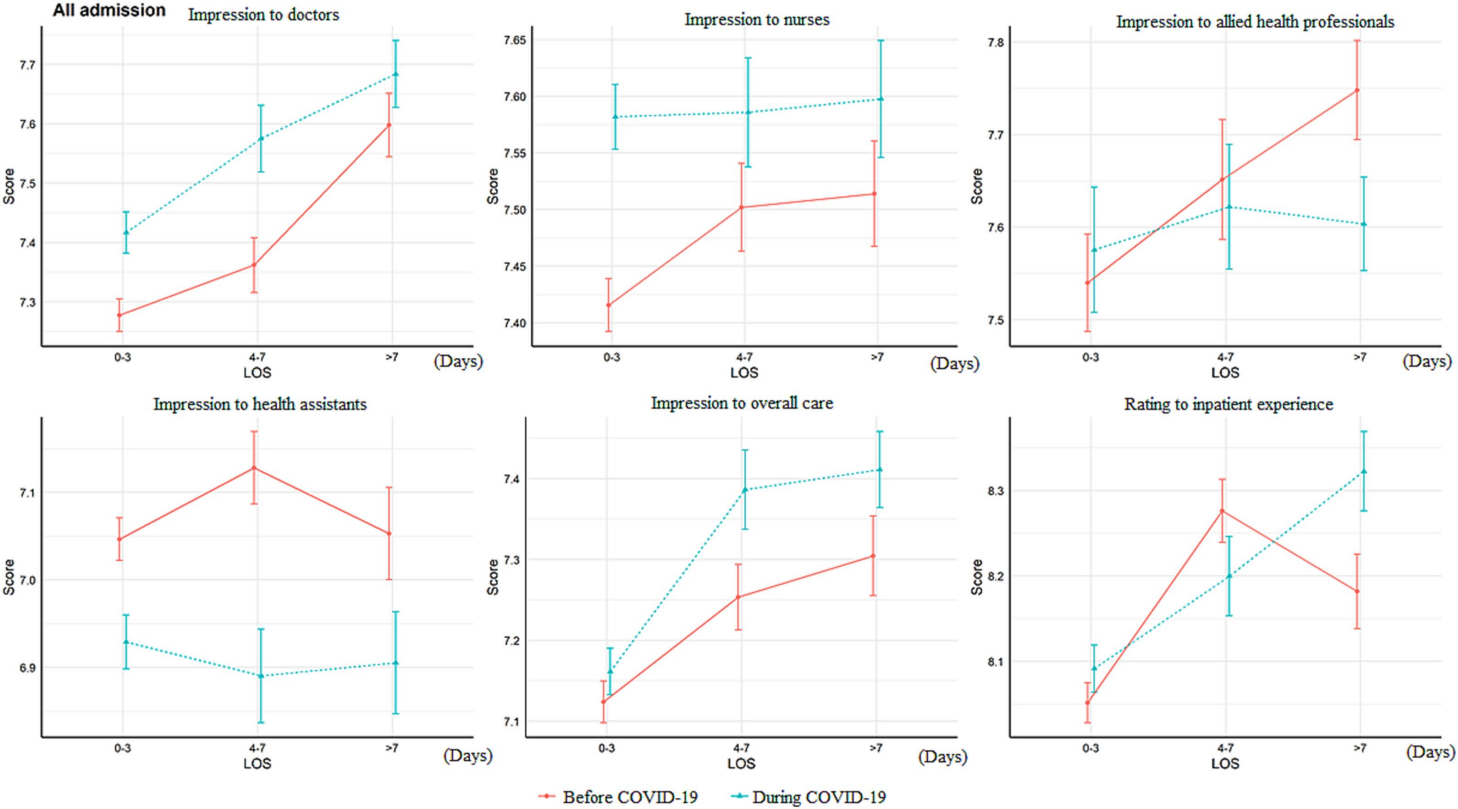
Difference of overall impression score change according to length of stay and before/during the pandemic LOS, length of stay. The Y-axis (“Score”) referred to the patient ratings to that item.

The perceived timeliness of hospital admissions was evaluated (Table 2). Before COVID-19, most of the participants (78.7%) felt that they did not had to wait for a long time to get admitted. This percentage was even higher during the pandemic (83.8%, *p* < 0.001). The percentage of those who” definitely” felt they waited for a long time decreased from 9.2% before the pandemic to 6.9% during the pandemic. The multiple regression analysis (Figure 1) also showed improvement in admission timeliness during the pandemic (adjusted odds ratio [AOR]: 1.60, 95% confidence interval [CI]: 1.40–1.82).

**Table 2:** Comparison of patient experience before and during the COVID-19 pandemic.

	Before COVID-19 pandemic	During COVID-19 pandemic	Total	*p* values
	*N* (%)	*N* (%)	*N* (%)	
Hospital admission				
Did you feel that you had to wait for a long time to get to ward and bed?				
No	4,587 (78.7)	3,284 (83.8)	7,871(80.8)	<0.001
Yes, to some extent	708 (12.2)	363 (9.3)	1,071 (11.0)	
Yes, definitely	533 (9.2)	272 (6.9)	805 (8.3)	
Hospital staff				
*Did you have confidence and trust in the doctors treating you?*				
No	118 (2.0)	96 (2.5)	214 (2.2)	0.021
Yes, sometimes	490 (8.5)	387 (9.9)	877 (9.1)	
Yes, always	5,169 (89.5)	3,430 (87.7)	8,599 (88.7)	
*Did you have confidence and trust in the nurses treating you?*				
No	45 (0.8)	45 (1.1)	90 (0.9)	0.001
Yes, sometimes	308 (5.3)	271 (6.9)	579 (5.9)	
Yes, always	5,503 (94.0)	3,628 (92.0)	9,131 (93.2)	
*Did you have confidence and trust in the allied health professionals treating you?*				
No	18 (1.0)	13 (1.2)	31 (1.0)	0.610
Yes, sometimes	70 (3.7)	46 (4.2)	116 (3.9)	
Yes, always	1,808 (95.4)	1,026 (94.6)	2,834 (95.1)	
*When you had important questions to ask a doctor, did your doctor provide a clear and understandable answer to you?*				
No	142 (2.7)	96 (2.8)	238 (2.7)	0.935
Yes, sometimes	719 (13.7)	483 (13.9)	1,202 (13.8)	
Yes, always	4,392 (83.6)	2,891 (83.3)	7,283 (83.5)	
*When you had important questions to ask a nurse, did the nurse provide a clear and understandable answer to you?*				
No	93 (1.7)	99 (2.9)	192 (2.2)	0.001
Yes, sometimes	508 (9.5)	300 (8.7)	808 (9.2)	
Yes, always	4,730 (88.7)	3,042 (88.4)	7,772 (88.6)	
*When you had important questions to ask an allied health professional, did they provide a clear and understandable answer to you?*				
No	8 (0.5)	6 (0.6)	14 (0.5)	0.161
Yes, sometimes	81 (4.6)	63 (6.2)	144 (5.2)	
Yes, always	1,672 (95.0)	946 (93.2)	2,618 (94.3)	
Patient care and treatment				
Did you have confidence in the decisions made about your care and treatment?				
No	147 (2.5)	101 (2.6)	248 (2.5)	0.880
Yes, sometimes	517 (8.8)	337 (8.5)	854 (8.7)	
Yes, always	5,192 (88.7)	3,506 (88.9)	8,698 (88.8)	
*Were you told the detailed aspects of your condition, treatment, operation or procedure and its results in a way you could understand?*				
No	241 (4.2)	183 (4.7)	424 (4.4)	<0.001
Yes, to some extent	724 (12.5)	698 (17.9)	1,422 (14.7)	
Yes, completely	4,820 (83.3)	3,011 (77.4)	7,831 (80.9)	
*Whenever you got worries or fears about your illness or the treatment, did the healthcare workers discuss/ comfort you about your condition?*				
No	537 (14.5)	714 (21.5)	1,251 (17.8)	<0.001
Yes, to some extent	367 (9.9)	612 (18.4)	979 (13.9)	
Yes, definitely	2,812 (75.7)	1,996 (60.1)	4,808 (68.3)	
*During admission, did you think the hospital staff have done everything they could to help control your pain?*				
No	66 (2.1)	59 (3.1)	125 (2.5)	<0.001
Yes, to some extent	117 (3.8)	310 (16.1)	427 (8.5)	
Yes, definitely	2,941 (94.1)	1,557 (80.8)	4,498 (89.1)	
*Were you involved in decisions about your care and treatment?*				
No	3,219 (56.4)	1,912 (51.4)	5,131 (54.4)	<0.001
Yes, to some extent	1,145 (20.1)	882 (23.7)	2027 (21.5)	
Yes, definitely	1,339 (23.5)	929 (25.0)	2,268 (24.1)	
Information on leaving hospital				
*Did a member of staff tell you in clear and understandable way on how to take your medications?*				
No	291 (6.1)	171 (5.2)	462 (5.7)	<0.001
Yes, to some extent	45 (0.9)	91 (2.8)	136 (1.7)	
Yes, completely	4,475 (93.0)	3,031 (92.0)	7,506 (92.6)	
*Did a member of staff explain to you the effect of the medications in a way you could understand?*				
No	314 (6.6)	198 (6.0)	512 (6.4)	<0.001
Yes, to some extent	60 (1.3)	120 (3.7)	180 (2.2)	
Yes, completely	4,408 (92.2)	2,961 (90.3)	7,369 (91.4)	
*Did a member of staff explain to you about medication side effects to watch for in a way you could understand?*				
No	1,205 (25.8)	1,036 (32.3)	2,241 (28.4)	<0.001
Yes, to some extent	173 (3.7)	206 (6.4)	379 (4.8)	
Yes, completely	3,293 (70.5)	1,970 (61.3)	5,263 (66.8)	
*Did a member of staff tell you about any danger signals you should watch for after you went home?*				
No	1,564 (29.7)	1,079 (30.5)	2,643 (30.0)	<0.001
Yes, to some extent	255 (4.8)	404 (11.4)	659 (7.5)	
Yes, completely	3,445 (65.4)	2,059 (58.1)	5,504 (62.5)	
*Did the hospital staff give your family or someone close to you all the information they needed in your care and recovery?*				
No	783 (28.0)	292 (30.9)	1,075 (28.7)	0.015
Yes, to some extent	491 (17.5)	189 (20.0)	680 (18.2)	
Yes, completely	1,526 (54.5)	464 (49.1)	1,990 (53.1)	
Overall impression				
*How would you rate the care you received from the doctors?*				
Very poor	17 (0.3)	19 (0.5)	36 (0.4)	<0.001
Poor	64 (1.1)	41 (1.1)	105 (1.1)	
Fair	1,018 (17.6)	691 (17.7)	1709 (17.6)	
Good	3,782 (65.5)	2,337 (59.7)	6,119 (63.2)	
Excellent/ Very good	896 (15.5)	825 (21.1)	1721 (17.8)	
*How would you rate the care you received form the nurses?*				
Very poor	8 (0.1)	13 (0.3)	21 (0.2)	<0.001
Poor	51 (0.9)	37 (0.9)	88 (0.9)	
Fair	695 (11.9)	417 (10.6)	1,112 (11.4)	
Good	4,359 (74.4)	2,820 (71.5)	7,179 (73.3)	
Excellent/ Very good	743 (12.7)	657 (16.7)	1,400 (14.3)	
*How would you rate the care you received from the allied health professionals?*				
Very poor	3 (0.2)	2 (0.2)	5 (0.2)	0.051
Poor	12 (0.6)	3 (0.3)	15 (0.5)	
Fair	157 (8.3)	83 (7.7)	240 (8.1)	
Good	1,421 (75.0)	861 (79.4)	2,282 (76.6)	
Excellent/ Very good	303 (16.0)	136 (12.5)	439 (14.7)	
*How would you rate the care you received from the healthcare assistants?*				
Very poor	19 (0.3)	18 (0.5)	37 (0.4)	<0.001
Poor	92 (1.6)	57 (1.5)	149 (1.5)	
Fair	1,189 (20.3)	1,099 (27.9)	2,288 (23.4)	
Good	4,152 (70.9)	2,451 (62.2)	6,603 (67.4)	
Excellent/ Very good	404 (6.9)	319 (8.1)	723 (7.4)	
*Overall, how would you rate the care you received?*				
Very poor	13 (0.2)	13 (0.3)	26 (0.3)	0.059
Poor	79 (1.4)	57 (1.5)	136 (1.4)	
Fair	1,068 (18.2)	632 (16.0)	1,700 (17.4)	
Good	4,135 (70.6)	2,861 (72.5)	6,996 (71.4)	
Excellent/Very good	561 (9.6)	381 (9.7)	942 (9.6)	
Total	5,856 (100.0)	3,944 (100.0)	9,800 (100.0)	

### 3.3. Hospital staff

Patients’ confidence and trust in the doctors reduced slightly before vs. during the COVID-19 pandemic (“always” had confidence and trust: 89.5 vs. 87.7%, *p* = 0.021), as shown in Table 2. The same was observed for patients’ confidence and trust in nurses (“always” had confidence and trust: 94.0 vs. 92.0%, *p* < 0.001), while there was no significant difference for allied health workers (*p* = 0.610). Regarding whether the staff provided clear and understandable answers to the important questions that the patients asked, more participants provided negative feedback for nurses during the pandemic (2.9 vs. 1.7%, *p* = 0.001), while the differences were not significant for doctors and allied health professionals. The multiple regression analysis (Figure 1) showed significant reductions in patients’ confidence in nurses (AOR: 0.78, 95% CI: 0.64–0.96) and responses for patient’s important questions provided by allied health workers (AOR: 0.49, 95% CI: 0.29–0.82) during the pandemic. Besides difference between before and during the pandemics, poorer self-rated health was associated with lower confidence in doctors, nurses, and care and treatment, and worse experience in receiving feedbacks from doctors and nurses for important questions (Supplementary Tables S1, S2).

### 3.4. Patient care and treatment

Different aspects of patient experience during care and treatment were measured (Table 2). While there was no significant difference in patients’ confidence in the hospital staff members’ decisions over care and treatment (*p* = 0.880), fewer participants, during the pandemic, reported that they were adequately informed of their conditions and treatment (77.4 vs. 83.3%, *p* < 0.001), received adequate comfort for their worries (60.1 vs. 75.7%, *p* < 0.001), and received help in relieving pain (80.8 vs. 94.1%, *p* < 0.001), compared with those admitted before the pandemic. After adjusting for demographics, health status, and hospitalization characteristics, it was also found that the following interactions with patients and responses to patient’s needs during care and treatment reduced during the pandemic (Figure 1): explanation regarding conditions and treatment (AOR: 0.82, 95% CI: 0.72–0.94), comfort for worries (AOR: 0.53, 95% CI: 0.46–0.61), and pain relief (AOR: 0.29, 95% CI: 0.22–0.37). The level of patient involvement in decisions regarding care and treatment during the pandemic was not significantly different from that before the pandemic (AOR: 1.06, 95% CI: 0.95–1.19).

### 3.5. Information during hospital discharge

Regarding information communication during hospital discharge (Table 2), communication between the staff and patients regarding the medications to take home reduced during the pandemic, especially regarding their side effects (61.3 vs. 70.5%, *p* < 0.001). Communication of information regarding the danger signals to notice (58.1 vs. 65.4%, *p* < 0.001) and that regarding care and recovery (49.1 vs. 54.5%, *p* = 0.015) also reduced during the pandemic. After adjusting for the covariates, communication of information on medication side effect reduced significantly during the pandemic (AOR: 0.72, 95% CI: 0.64–0.82), while there were marginally significant reductions in information communication on medication effect (AOR: 0.82, 95% CI: 0.66–1.02) and danger signals to notice (AOR: 0.91, 95% CI: 0.81–1.02; Figure 1).

### 3.6. Overall impression

**Figure 2:**
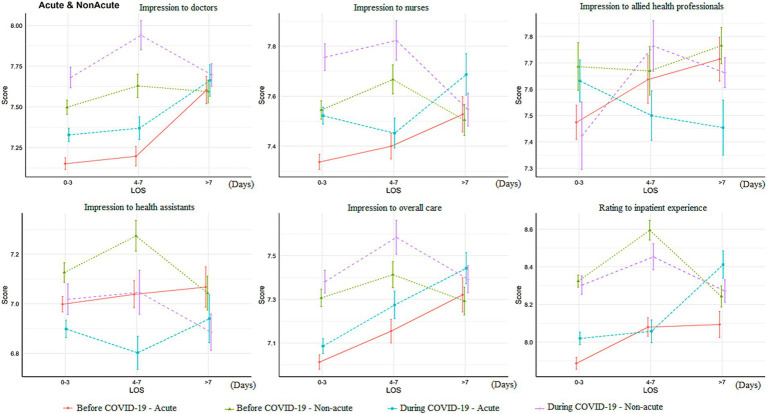
Difference of overall impression score change according to admission source of the patients LOS, length of stay. The Y-axis (“Score”) referred to the patient ratings to that item.

**Figure 3:**
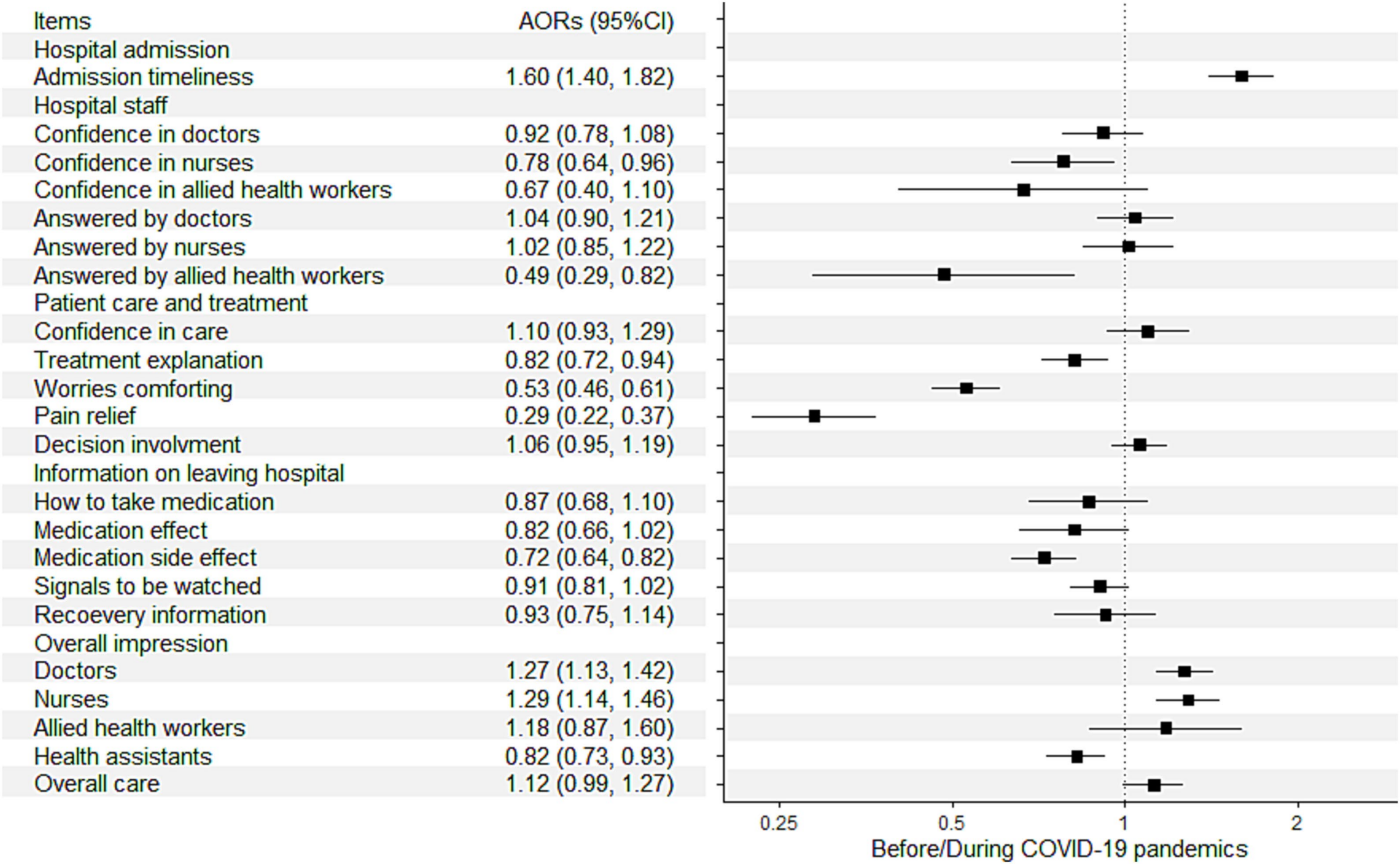
Association between patient experience and before/during the pandemic with adjustment of covariates AOR, adjusted odds ratio; CI, confidence interval. This figure showed the ordinal logistic regression outcomes for the association between patient experience and before/during the pandemics, where AOR > 1 referred to higher rating to that item during the pandemics and AOR < 1 referred to lower rating during the pandemics.

In contrast to the decreased satisfaction with multiple aspects of the patient journey, the overall impression rating of doctors (excellent/very good: 21.1 vs. 15.5%, *p* < 0.001) and nurses increased during the pandemic (excellent/very good: 16.7 vs. 12.7%, *p* < 0.001; Table 2). However, the rating of healthcare assistants decreased during the pandemic (good: 62.2 vs. 70.9%; fair: 27.9 vs. 20.3%, *p* < 0.001). There was no significant difference in the rating of the care they received (*p* = 0.059). The two-way ANOVA results showed that the ratings of doctors (*p* < 0.001), nurses (*p* < 0.001), and overall care (*p* = 0.027) were significantly higher during the pandemic, but the rating of health assistants (*p* < 0.001) was lower (Figure 2). Patients admitted for non-acute reasons tended to give higher ratings to different hospital staff members and overall care than those admitted for acute reasons (Figure 3). The ratings of doctors, nurses, and overall care were higher during the pandemic in both acutely admitted (doctors: *p* < 0.001; nurses: *p* < 0.001; overall: *p* = 0.029) and non-acutely admitted patients (doctors: *p* < 0.001; nurses: *p* = 0.001; overall: *p* = 0.027), while the rating of health assistants was lower in both groups of patients (acute, *p* < 0.001; non-acute, *p* = 0.004).

The multiple regression analysis (Figure 1) showed similar results: the overall rating of doctors (AOR: 1.27, 95% CI: 1.13–1.42) and nurses (AOR: 1.29, 95% CI: 1.14–1.46) increased, while the rating of health assistants (AOR: 0.82, 95% CI: 0.99–1.27) reduced during the pandemic. There was a marginally significant increase in the rating of overall care during the pandemic (AOR: 1.12, 95% CI: 0.99–1.27). It was also found that poorer self-rated health was consistently associated with lower overall rating of all disciplines of healthcare workers (Supplementary Table S5).

## 4. Discussion

This study explored the impact on patient experience due the COVID-19 pandemic and examined the changes of patient experience in multiple dimensions throughout the inpatient journey in public hospitals when compared to pre-pandemic situation, using data collected from a random sample of Chinese patients in HK with a well-validated survey tool. The survey findings indicated reductions in patients’ confidence in and responsiveness of hospital staff, communication of information regarding care and treatment, and communication of information at hospital discharge during the pandemic, as well as the increase in overall impression rating of doctors and nurses. The findings are important for the service providers and staff to understand where improvement and caution are required, and can be used to develop strategies aimed at supporting the staff, to provide better care to the patients in the new normal or prepare for the new pandemics in coming future.

In a number of dimensions, this study reported poorer patient experience or worsened performance during the pandemic, compared to the pre-pandemic period. This decreasing trend is particularly substantial in the communication regarding care and treatment dimension. The staff were reported to be less responsive to patients’ needs for knowledge of their condition and treatment, patients’ emotional needs (comfort for worries and fears), and their requests to reduce physical discomfort. The reduced responsiveness of the staff was also reflected by the reduced responses from allied health workers to the patients’ questions and reduced information communication regarding take-home medications. This reduction in responsiveness may be partially due to the increased use of PPE among the staff providing service which affected their ability to communicate (12) and finally increased the fear and anxiety from the patients (13). A study in the UK reported that 37% of the participants reported that it was “sometimes,” “often” or “always” difficult to communicate with staff wearing PPE (21). Another possible reason could be that healthcare workers were more likely than the general public to experience mental health problems, including stress, fatigue, or burnout during the pandemic (24, 25), which affects their awareness and willingness to be as responsive in service provision as usual. Increasing workload in healthcare provision during COVID-19 pandemic definitely affects the time that healthcare workers can allot to the communication and information dissemination to patients and their caregivers, which are important to both of them due to the disconnection once the patients stayed in the hospital (14). Particularly, it may have a bigger impact on the quality of nurse–patient relationship since the nurse who plays a key role in the direct patient care. It may be a possible reason to explain the reduction in confidence on nurse in comparison with the pre-pandemic period.

Nevertheless, patient experience improved in a few dimensions. The perceived timeliness of hospital admission increased during the pandemic. This improvement was probably due to decreased utilization of hospital services, including planned visits and emergency department visits, as well as general hospital admissions (26, 27), which enabled the patients to gain access to hospital services more quickly. Also, the admission procedure in term of bed assignment and administrative procedure was speed up with the aim to reduce crowd upon the admission site during COVID-19 pandemic. Meanwhile, the overall impression rating of doctors, nurses, and overall care increased after COVID-19 outbreak, despite the patients’ experiences in other dimensions. The improved ratings of doctors, nurses, and care were possibly due to the anchoring effect (28) or “gratitude bias” (20), whereby patients’ evaluation of their experience could be influenced by their reduced expectations of the staff due to the pandemic, causing them to give a relatively higher rating to express their “gratitude” to the doctors and nurses for their work during this difficult period. However, the patients seem to be more tolerant to doctors and nurses than to health assistants, as the rating for the health assistants during the pandemic was lower than that before the pandemic. It is possible that a different standard applied to health assistants from doctors and nurses. Another possible reason could be that health assistants were not be adequately trained to cope with the shifted context of healthcare service provision during the pandemic, which made them perform worse than before.

Interestingly, the overall patient experience was high in both before and during COVID-19 pandemic, which was similar to the findings from the NHS (20). This might be due to the recognition and appreciation of healthcare workers’ efforts, with the mounting demands and challenges during the pandemic. Patient experience differed according to their admission source both before and after the pandemic. For overall impression ratings, patients admitted for non-acute reasons, including planned admission or hospital transfer, gave higher ratings to different staff and overall care than those admitted for acute reasons. The differences before and during the pandemics in the ratings were consistent across admission sources, as there were no interaction effects. Regarding their experience in different dimensions (Supplementary Tables S1–S5), non-acutely admitted patients had equal or better experience in their patient journey than acutely admitted patients, except with the communication on take-home medications dimension, while this disparity was smaller during the pandemic for the explanation for patient’s condition and treatment dimension. Again, this finding is similar to those of a patient experience survey in the UK (20), which suggested that it was more challenging to provide satisfying services to acute inpatients than to non-acute inpatients, since there might be more uncertainty in dealing with their condition and administrative processes. Although acutely admitted inpatients were more likely to experience a worse journey than the others, this disparity was not amplified during the pandemic, despite the additional requirement for infection control at admission. This could be due to the reduced number of patients at the emergency department (26) and a clear guideline created for infection control and clinical procedures for emergency departments and inpatient wards (29).

Self-perceived health was also found to be associated with patient experience (Supplementary Tables S1–S5). Patients with worse self-perceived health reported worse experience regarding confidence in and communication with the hospital staff as well as the overall impression during their inpatient journey, which is similar to the results of several previous studies that better self-rated health is associated with greater satisfaction with healthcare services (30, 31). One of the studies (31) suggested that patient satisfaction was not only associated with quality of care but also to the well-being of the patients, which implied that this factor should be considered when comparing the patient experience or satisfaction of different populations. However, the latest communication technologies such as telemedicine service Apps, Communication Apps may provide us a new direction to build the bridge between the gaps and fulfil the need of patients and their caregivers and improve patients’ health outcomes. Also, it may help to reduce the workload of healthcare staff in the new normal or preparing for the next pandemics (32–34). In parallel, more studies are needed to explore the concerns and difficulties from patients or caregivers for their self-care management due to their limited health literacy (35, 36).

This study had some limitations. First, this is a cross-sectional study, implying that no causal relationship between the COVID-19 outbreak and patient experience can be found. Second, a few health and medical care information regarding patient diagnosis and the department they were admitted into was not made available to the research team by the service providers out of the consideration of privacy protection; therefore, these variables were missing from the analysis, and LOS, self-rated health, and admission source were included as covariates. Third, the cut-off time point selected to define the pandemic period could have an impact on the result, which is why another time point, 4 January 2020 when emergency response level was raised from “alert” to “serious,” was selected for a sensitivity analysis (Supplementary Table S6). This sensitivity analysis showed similar findings to those reported in the above section, which suggested that the results are robust. In order to respond the changes of patient experience during the pandemic, further patient and staff focus group discussion would need to enrich the existing measurement tool for the patient experience. Moreover, repeated cross-sectional surveys should be kept at different time points in the future. It will help to monitor the changes in patient experience and provide useful information for policymakers to improve the quality of care under the new normal.

## 5. Conclusion

In summary, the patients hospitalized during the pandemic reported similar or worse responsiveness in most of the dimensions of their patient journey than those admitted before the pandemic, which may be due to infection control measures and mental health problems of the healthcare workers. The patients gave higher overall impression ratings to doctors, nurses, and overall care, but a lower rating to health assistants, which implied a higher level of patient respect or gratitude to doctors and nurses than to health assistants, and possible insufficient training of health assistants’ and inability to cope with the particular situation in pandemics. A clearer procedure and guidelines should be considered for healthcare workers to communicate with patients. Adequate training and consultants for mental problems should also be made available to health assistants and other staff who are less experienced and less professional in dealing with medical care during a pandemic.

## Data availability statement

The original contributions presented in the study are included in the article/Supplementary material, further inquiries can be directed to the corresponding author.

## Ethics statement

The studies involving humans were approved by Hospital Authority Clinical Research Ethics Committees. The studies were conducted in accordance with the local legislation and institutional requirements. The ethics committee/institutional review board waived the requirement of written informed consent for participation from the participants or the participants’ legal guardians/next of kin because it is a telephone survey and verbal informed consent was confirmed before the interview.

## Author contributions

ELYW and EKY conceived the study design. ELYW was the project in-charge to lead the study. KW and AWLC extracted the data and conducted the analysis with input from all authors. CG provided advice on the interpretation of the findings. ELYW and KW drafted the manuscript and all authors edited the manuscript. All authors read and approved the final manuscript.

## Conflict of interest

The authors declare that the research was conducted in the absence of any commercial or financial relationships that could be construed as a potential conflict of interest.

## Publisher’s note

All claims expressed in this article are solely those of the authors and do not necessarily represent those of their affiliated organizations, or those of the publisher, the editors and the reviewers. Any product that may be evaluated in this article, or claim that may be made by its manufacturer, is not guaranteed or endorsed by the publisher.
