# Supplementary material for: Thinking beyond the virus: perspective of patients on the quality of hospital care before and during the COVID-19 pandemic
Source: Front Public Health. 2023 Sep 8;11:1152054. doi: 10.3389/fpubh.2023.1152054 (PMC10515219; doi:10.3389/fpubh.2023.1152054)
Supplement: Supplementary file 1 [file Table_1.DOCX]

# Appendix

## ***Table S1 – Association between admission timeliness/confidence and various factors***

|  | Admission timeliness | | Confidence in doctors | | Confidence in nurses | | Confidence in allied health professionals | | Confidence in care and treatment | |
| --- | --- | --- | --- | --- | --- | --- | --- | --- | --- | --- |
| **Ordinal logit model** | **AOR** | **95%CI** | **AOR** | **95%CI** | **AOR** | **95%CI** | **AOR** | **95%CI** | **AOR** | **95%CI** |
| **Age group (18-40 as reference)** | | |  |  |  |  |  |  |  |  |
| 41-60 | 1.15* | (1.00, 1.33) | 1.14 | (0.96, 1.36) | 1.13 | (0.91, 1.41) | 1.18 | (0.70, 1.99) | 1.16 | (0.97, 1.37) |
| 61-70 | 1.21* | (1.03, 1.41) | 1.66** | (1.37, 2.03) | 1.51* | (1.18, 1.93) | 1.39 | (0.80, 2.42) | 1.55** | (1.28, 1.88) |
| 71+ | 1.24* | (1.02, 1.52) | 1.91** | (1.48, 2.45) | 1.55* | (1.15, 2.10) | 1.79 | (0.91, 3.49) | 2.14** | (1.65, 2.78) |
| **Female** | 0.82** | (0.74, 0.92) | 0.92 | (0.81, 1.04) | 0.82* | (0.70, 0.96) | 1.28 | (0.91, 1.80) | 0.81* | (0.71, 0.92) |
| **After COVID-19 outbreak** | 1.60** | (1.40, 1.82) | 0.92 | (0.78, 1.08) | 0.78* | (0.64, 0.96) | 0.67 | (0.40, 1.10) | 1.10 | (0.93, 1.29) |
| **Length of stay (LOS, 1-3 days as reference)** | | |  |  |  |  |  |  |  |  |
| 4-7 days | 1.21 | (1.00, 1.46) | 1.44* | (1.11, 1.86) | 1.10 | (0.81, 1.50) | 1.57 | (0.83, 2.99) | 1.39* | (1.09, 1.78) |
| >7 days | 1.19 | (0.95, 1.47) | 1.21 | (0.94, 1.57) | 0.97 | (0.72, 1.32) | 1.00 | (0.60, 1.66) | 1.40* | (1.08, 1.82) |
| **Interaction: COVID*LOS** |  |  |  |  |  |  |  |  |  |  |
| COVID*4-7 days | 1.22 | (0.87, 1.72) | 0.77 | (0.53, 1.13) | 0.95 | (0.60, 1.50) | 2.12 | (0.71, 6.37) | 0.93 | (0.63, 1.38) |
| COVID*>7 days | 1.20 | (0.79, 1.84) | 1.19 | (0.78, 1.82) | 1.01 | (0.63, 1.63) | 1.65 | (0.72, 3.81) | 0.96 | (0.62, 1.48) |
| **Admitted from OPD/transferring (A&E as reference)** | 7.39** | (5.99, 9.11) | 1.92** | (1.56, 2.35) | 1.62** | (1.28, 2.06) | 1.60 | (0.99, 2.58) | 1.96** | (1.60, 2.39) |
| **Interaction: COVID*Admission source** | | |  |  |  |  |  |  |  |  |
| OVID*OPD/transferring | 0.95 | (0.65, 1.40) | 0.93 | (0.69, 1.27) | 0.89 | (0.62, 1.28) | 0.93 | (0.43, 2.01) | 1.09 | (0.79, 1.50) |
| **Self-rated health (Very good as reference)** |  |  |  |  |  |  |  |  |  |  |
| Good | 1.02 | (0.73, 1.42) | 1.20 | (0.78, 1.85) | 0.68 | (0.34, 1.35) | 0.74 | (0.17, 3.17) | 1.06 | (0.67, 1.67) |
| Fair | 1.01 | (0.72, 1.40) | 0.61* | (0.40, 0.93) | 0.38* | (0.19, 0.75) | 0.53 | (0.12, 2.24) | 0.57* | (0.36, 0.90) |
| Poor | 1.27 | (0.86, 1.89) | 0.33** | (0.21, 0.53) | 0.20** | (0.10, 0.41) | 0.35 | (0.08, 1.58) | 0.28** | (0.17, 0.45) |
| Very poor | 1.50 | (0.72, 3.14) | 0.37* | (0.18, 0.74) | 0.24* | (0.09, 0.61) | 0.45 | (0.06, 3.41) | 0.28** | (0.14, 0.55) |

Note: *P<0.05, **P<0.001; Random effect applied to account for difference across hospitals; AOR: adjusted odds ratio for one unit improvement in the question scale.

## ***Table S2. Association between responsiveness of different healthcare workers to important questions asked by the patients and various factors***

|  | Doctors |  |  | Nurses |  |  | Allied health professionals | |
| --- | --- | --- | --- | --- | --- | --- | --- | --- |
| **Ordinal logit model** | **AOR** | **95%CI** |  | **AOR** | **95%CI** |  | **AOR** | **95%CI** |
| **Age group (18-40 as reference)** |  |  |  |  |  |  |  |  |
| 41-60 | 0.92 | (0.78, 1.08) |  | 1.01 | (0.83, 1.22) |  | 1.50 | (0.91, 2.48) |
| 61-70 | 1.12 | (0.94, 1.34) |  | 0.97 | (0.79, 1.20) |  | 1.46 | (0.87, 2.47) |
| 71+ | 1.15 | (0.92, 1.45) |  | 0.97 | (0.75, 1.24) |  | 2.09* | (1.09, 4.03) |
| **Female** | 0.98 | (0.88, 1.10) |  | 0.87* | (0.76, 0.99) |  | 1.08 | (0.77, 1.50) |
| **After COVID-19 outbreak** | 1.04 | (0.90, 1.21) |  | 1.02 | (0.85, 1.22) |  | 0.49* | (0.29, 0.82) |
| **Length of stay (LOS, 1-3 days as reference)** |  |  |  |  |  |  |  |  |
| 4-7 days | 1.23 | (1.00, 1.52) |  | 0.95 | (0.75, 1.21) |  | 1.10 | (0.61, 1.98) |
| >7 days | 1.12 | (0.90, 1.39) |  | 0.75* | (0.60, 0.94) |  | 0.99 | (0.59, 1.68) |
| **Interaction: COVID*LOS** |  |  |  |  |  |  |  |  |
| COVID*4-7 days | 0.97 | (0.69, 1.36) |  | 1.00 | (0.69, 1.46) |  | 1.27 | (0.52, 3.13) |
| COVID*>7 days | 1.06 | (0.74, 1.51) |  | 1.17 | (0.79, 1.72) |  | 1.08 | (0.48, 2.43) |
| **Admitted from OPD/transferring (A&E as reference)** | 1.76** | (1.48, 2.10) |  | 1.60** | (1.33, 1.92) |  | 1.00 | (0.62, 1.62) |
| **Interaction: COVID*Admission source** |  |  |  |  |  |  |  |  |
| COVID*OPD/transferring | 0.92 | (0.70, 1.21) |  | 0.75 | (0.56, 1.02) |  | 1.92 | (0.92, 3.98) |
| **Self-rated health (Very good as reference)** |  |  |  |  |  |  |  |  |
| Good | 0.70 | (0.44, 1.09) |  | 0.46* | (0.24, 0.87) |  | 0.27 | (0.04, 2.00) |
| Fair | 0.43** | (0.27, 0.67) |  | 0.32* | (0.17, 0.61) |  | 0.26 | (0.04, 1.97) |
| Poor | 0.31** | (0.19, 0.50) |  | 0.25** | (0.13, 0.49) |  | 0.11* | (0.01, 0.83) |
| Very poor | 0.28** | (0.14, 0.55) |  | 0.25* | (0.10, 0.60) |  | 0.11 | (0.01, 1.06) |

Note: *P<0.05, **P<0.001; Random effect applied to account for difference across hospitals; AOR: adjusted odds ratio for one unit improvement in the question scale.

## ***Table S3. Association between responsiveness during patient treatment and care and various factors***

|  | Treatment explanation | | Worries comforting | | Pain relief | | Decision involvement | |
| --- | --- | --- | --- | --- | --- | --- | --- | --- |
| **Ordinal logit model** | **AOR** | **95%CI** | **AOR** | **95%CI** | **AOR** | **95%CI** | **AOR** | **95%CI** |
| **Age group (18-40 as reference)** |  |  |  |  |  |  |  |  |
| 41-60 | 0.93 | (0.80, 1.08) | 0.88 | (0.76, 1.01) | 1.07 | (0.83, 1.37) | 0.63** | (0.56, 0.70) |
| 61-70 | 0.95 | (0.81, 1.12) | 1.09 | (0.93, 1.27) | 1.11 | (0.84, 1.46) | 0.42** | (0.37, 0.47) |
| 71+ | 0.78* | (0.64, 0.94) | 1.18 | (0.97, 1.43) | 1.12 | (0.79, 1.60) | 0.22** | (0.19, 0.26) |
| **Female** | 0.87* | (0.79, 0.97) | 0.96 | (0.87, 1.07) | 0.92 | (0.76, 1.10) | 0.85** | (0.79, 0.92) |
| **After COVID-19 outbreak** | 0.82* | (0.72, 0.94) | 0.53** | (0.46, 0.61) | 0.29** | (0.22, 0.37) | 1.06 | (0.95, 1.19) |
| **Length of stay (LOS, 1-3 days as reference)** |  |  |  |  |  |  |  |  |
| 4-7 days | 1.40* | (1.13, 1.73) | 1.38* | (1.11, 1.70) | 1.77* | (1.11, 2.82) | 1.07 | (0.93, 1.23) |
| >7 days | 0.99 | (0.81, 1.21) | 1.53** | (1.24, 1.90) | 1.26 | (0.83, 1.90) | 1.25* | (1.08, 1.45) |
| **Interaction: COVID*LOS** |  |  |  |  |  |  |  |  |
| COVID*4-7 days | 0.77 | (0.57, 1.04) | 0.85 | (0.63, 1.13) | 1.06 | (0.59, 1.92) | 1.01 | (0.80, 1.27) |
| COVID*>7 days | 1.11 | (0.82, 1.51) | 1.03 | (0.76, 1.39) | 1.40 | (0.81, 2.41) | 1.01 | (0.80, 1.28) |
| **Admitted from OPD/transferring (A&E as reference)** | 2.37** | (2.00, 2.80) | 1.51** | (1.28, 1.78) | 1.49* | (1.05, 2.10) | 1.07 | (0.95, 1.20) |
| **Interaction: COVID*Admission source** |  |  |  |  |  |  |  |  |
| COVID*OPD/transferring | 0.69* | (0.54, 0.88) | 1.06 | (0.84, 1.33) | 0.85 | (0.55, 1.31) | 1.21* | (1.02, 1.45) |
| **Self-rated health (Very good as reference)** |  |  |  |  |  |  |  |  |
| Good | 1.18 | (0.84, 1.65) | 1.01 | (0.74, 1.37) | 0.53 | (0.26, 1.09) | 0.56** | (0.43, 0.72) |
| Fair | 0.81 | (0.58, 1.12) | 0.89 | (0.66, 1.21) | 0.47* | (0.23, 0.95) | 0.72* | (0.56, 0.93) |
| Poor | 0.54* | (0.37, 0.78) | 0.88 | (0.62, 1.26) | 0.37* | (0.17, 0.81) | 1.02 | (0.76, 1.36) |
| Very poor | 0.44* | (0.25, 0.77) | 0.84 | (0.48, 1.48) | 0.46 | (0.15, 1.44) | 1.39 | (0.87, 2.22) |

Note: *P<0.05, **P<0.001; Random effect applied to account for difference across hospitals; AOR: adjusted odds ratio for one unit improvement in the question scale.

## ***Table S4. Association between provision of information at discharge and care and various factors***

|  | How to take medication | | Medication effectiveness | | Medication side effect | | Danger signal to be watched | | Recovery information | |
| --- | --- | --- | --- | --- | --- | --- | --- | --- | --- | --- |
| **Ordinal logit model** | **AOR** | **95%CI** | **AOR** | **95%CI** | **AOR** | **95%CI** | **AOR** | **95%CI** | **AOR** | **95%CI** |
| **Age group (18-40 as reference)** | | |  |  |  |  |  |  |  |  |
| 41-60 | 1.11 | (0.88, 1.41) | 1.10 | (0.89, 1.37) | 0.98 | (0.85, 1.12) | 0.89 | (0.78, 1.01) | 0.97 | (0.80, 1.18) |
| 61-70 | 1.18 | (0.91, 1.53) | 1.33* | (1.04, 1.70) | 1.13 | (0.97, 1.30) | 0.94 | (0.82, 1.08) | 1.41* | (1.15, 1.72) |
| 71+ | 0.94 | (0.68, 1.29) | 1.05 | (0.78, 1.42) | 1.31* | (1.09, 1.57) | 1.12 | (0.95, 1.32) | 1.59** | (1.27, 2.00) |
| **Female** | 0.84 | (0.71, 1.00) | 0.89 | (0.76, 1.05) | 0.94 | (0.85, 1.03) | 0.87* | (0.80, 0.95) | 1.03 | (0.91, 1.17) |
| **After COVID-19 outbreak** | 0.87 | (0.68, 1.10) | 0.82 | (0.66, 1.02) | 0.72** | (0.64, 0.82) | 0.91 | (0.81, 1.02) | 0.93 | (0.75, 1.14) |
| **Length of stay (LOS, 1-3 days as reference)** | | |  |  |  |  |  |  |  |  |
| 4-7 days | 1.89** | (1.34, 2.67) | 1.87** | (1.34, 2.59) | 1.36* | (1.14, 1.63) | 1.49** | (1.27, 1.75) | 1.46** | (1.20, 1.77) |
| >7 days | 3.11** | (2.06, 4.68) | 2.67** | (1.84, 3.88) | 1.54** | (1.28, 1.86) | 1.71** | (1.44, 2.04) | 1.95** | (1.59, 2.39) |
| **Interaction: COVID*LOS** |  |  |  |  |  |  |  |  |  |  |
| COVID*4-7 days | 1.15 | (0.66, 1.98) | 1.05 | (0.64, 1.73) | 0.87 | (0.67, 1.14) | 0.89 | (0.69, 1.14) | 0.96 | (0.64, 1.42) |
| COVID*>7 days | 1.45 | (0.74, 2.84) | 1.53 | (0.84, 2.82) | 0.99 | (0.74, 1.31) | 1.04 | (0.80, 1.36) | 1.04 | (0.70, 1.54) |
| **Admitted from OPD/transferring (A&E as reference)** | 0.55** | (0.43, 0.70) | 0.62** | (0.49, 0.79) | 1.02 | (0.88, 1.18) | 1.64** | (1.43, 1.87) | 1.52** | (1.30, 1.78) |
| **Interaction: COVID*Admission source** | | |  |  |  |  |  |  |  |  |
| COVID*OPD/transferring | 0.99 | (0.69, 1.42) | 0.93 | (0.66, 1.31) | 1.07 | (0.86, 1.32) | 0.88 | (0.73, 1.07) | 0.84 | (0.62, 1.15) |
| **Self-rated health (Very good as reference)** | | |  |  |  |  |  |  |  |  |
| Good | 1.13 | (0.69, 1.84) | 1.17 | (0.74, 1.83) | 1.36* | (1.00, 1.84) | 0.95 | (0.72, 1.26) | 1.11 | (0.67, 1.82) |
| Fair | 1.58 | (0.97, 2.59) | 1.52 | (0.97, 2.40) | 1.09 | (0.80, 1.47) | 0.84 | (0.63, 1.11) | 0.69 | (0.42, 1.14) |
| Poor | 1.58 | (0.86, 2.88) | 1.31 | (0.76, 2.25) | 0.81 | (0.57, 1.14) | 0.71* | (0.52, 0.98) | 0.65 | (0.38, 1.12) |
| Very poor | 1.97 | (0.55, 7.00) | 2.36 | (0.67, 8.23) | 0.70 | (0.39, 1.24) | 0.64 | (0.38, 1.08) | 0.56 | (0.25, 1.27) |

Note: *P<0.05, **P<0.001; Random effect applied to account for difference across hospitals; AOR: adjusted odds ratio for one unit improvement

## ***Table S5. Association between impression to different healthcare workers and overall care and various factors***

|  | Doctors | | Nurses | | Allied health professionals | | Health assistant | | Overall care | |
| --- | --- | --- | --- | --- | --- | --- | --- | --- | --- | --- |
| **Ordinal logit model** | **AOR** | **95%CI** | **AOR** | **95%CI** | **AOR** | **95%CI** | **AOR** | **95%CI** | **AOR** | **95%CI** |
| **Age group (18-40 as reference)** | | |  |  |  |  |  |  |  |  |
| 41-60 | 0.99 | (0.88, 1.12) | 0.90 | (0.79, 1.02) | 1.03 | (0.76, 1.38) | 0.86* | (0.76, 0.98) | 0.93 | (0.82, 1.06) |
| 61-70 | 1.12 | (0.99, 1.27) | 0.80* | (0.70, 0.92) | 0.90 | (0.67, 1.22) | 0.80* | (0.70, 0.92) | 1.08 | (0.94, 1.24) |
| 71+ | 1.08 | (0.93, 1.26) | 0.78* | (0.66, 0.92) | 0.90 | (0.64, 1.26) | 0.78* | (0.67, 0.92) | 1.11 | (0.94, 1.32) |
| **Female** | 0.89* | (0.82, 0.97) | 0.85** | (0.78, 0.93) | 1.19 | (1.00, 1.41) | 0.81** | (0.74, 0.88) | 0.92 | (0.85, 1.01) |
| **After COVID-19 outbreak** | 1.27** | (1.13, 1.42) | 1.29** | (1.14, 1.46) | 1.18 | (0.87, 1.60) | 0.82* | (0.73, 0.93) | 1.12 | (0.99, 1.27) |
| **Length of stay (LOS, 1-3 days as reference)** | | |  |  |  |  |  |  |  |  |
| 4-7 days | 1.26* | (1.09, 1.46) | 1.29* | (1.10, 1.51) | 1.44* | (1.08, 1.92) | 1.26* | (1.08, 1.46) | 1.34** | (1.15, 1.57) |
| >7 days | 1.76** | (1.51, 2.05) | 1.37** | (1.16, 1.62) | 1.40* | (1.07, 1.82) | 1.23* | (1.04, 1.44) | 1.64** | (1.39, 1.94) |
| **Interaction: COVID*LOS** |  |  |  |  |  |  |  |  |  |  |
| COVID*4-7 days | 1.07 | (0.85, 1.35) | 0.85 | (0.66, 1.10) | 0.76 | (0.47, 1.21) | 0.80 | (0.63, 1.02) | 1.12 | (0.87, 1.44) |
| COVID*>7 days | 0.74* | (0.58, 0.94) | 0.77 | (0.59, 1.00) | 0.73 | (0.47, 1.13) | 0.81 | (0.63, 1.04) | 0.90 | (0.69, 1.18) |
| **Admitted from OPD/transferring (A&E as reference)** | 1.44** | (1.28, 1.63) | 1.26** | (1.11, 1.43) | 1.15 | (0.90, 1.47) | 1.19* | (1.05, 1.35) | 1.41** | (1.24, 1.60) |
| **Interaction: COVID*Admission source** | | |  |  |  |  |  |  |  |  |
| COVID*OPD/transferring | 1.11 | (0.92, 1.33) | 1.10 | (0.91, 1.34) | 0.86 | (0.58, 1.27) | 1.02 | (0.85, 1.23) | 1.05 | (0.87, 1.28) |
| **Self-rated health (Very good as reference)** | | |  |  |  |  |  |  |  |  |
| Good | 0.40** | (0.30, 0.52) | 0.40** | (0.31, 0.53) | 0.56 | (0.31, 1.00) | 0.50** | (0.37, 0.67) | 0.45** | (0.34, 0.60) |
| Fair | 0.26** | (0.19, 0.34) | 0.29** | (0.22, 0.37) | 0.37* | (0.21, 0.66) | 0.38** | (0.29, 0.51) | 0.26** | (0.20, 0.35) |
| Poor | 0.16** | (0.12, 0.22) | 0.18** | (0.13, 0.25) | 0.24** | (0.13, 0.46) | 0.27** | (0.19, 0.37) | 0.12** | (0.09, 0.17) |
| Very poor | 0.13** | (0.08, 0.22) | 0.12** | (0.07, 0.20) | 0.65 | (0.26, 1.65) | 0.24** | (0.14, 0.40) | 0.11** | (0.06, 0.18) |

Note: *P<0.05, **P<0.001; Random effect applied to account for difference across hospitals; AOR: adjusted odds ratio for one unit improvement

## ***Table S6. Sensitivity analysis using different cut-off day for COVID-19 outbreaks: association between impression to different healthcare workers and overall care and various factors***

| **How would you rate the care from…?** | Doctors | | Nurses | | Allied health professionals | | Health assistant | | Overall care | |
| --- | --- | --- | --- | --- | --- | --- | --- | --- | --- | --- |
|  | **AOR** | **95%CI** | **AOR** | **95%CI** | **AOR** | **95%CI** | **AOR** | **95%CI** | **AOR** | **95%CI** |
| **Age group (18-40 as reference)** | | |  |  |  |  |  |  |  |  |
| 41-60 | 0.99 | (0.88, 1.11) | 0.90 | (0.79, 1.02) | 1.02 | (0.76, 1.37) | 0.87* | (0.77, 0.98) | 0.93 | (0.82, 1.06) |
| 61-70 | 1.11 | (0.98, 1.26) | 0.80* | (0.70, 0.92) | 0.89 | (0.66, 1.21) | 0.80* | (0.70, 0.92) | 1.08 | (0.94, 1.24) |
| 71+ | 1.08 | (0.93, 1.26) | 0.78* | (0.66, 0.92) | 0.89 | (0.63, 1.24) | 0.78* | (0.67, 0.92) | 1.12 | (0.95, 1.32) |
| **Female** | 0.89* | (0.82, 0.96) | 0.85* | (0.78, 0.93) | 1.19* | (1.00, 1.41) | 0.81** | (0.74, 0.88) | 0.93 | (0.85, 1.01) |
| **After COVID-19 outbreak** | 1.24** | (1.10, 1.39) | 1.28** | (1.13, 1.45) | 1.17 | (0.87, 1.57) | 0.83* | (0.74, 0.93) | 1.13* | (1.00, 1.28) |
| **Length of stay (LOS, 1-3 days as reference)** | | |  |  |  |  |  |  |  |  |
| 4-7 days | 1.28* | (1.10, 1.49) | 1.24* | (1.05, 1.47) | 1.49* | (1.10, 2.01) | 1.24* | (1.06, 1.46) | 1.33* | (1.12, 1.57) |
| >7 days | 1.70** | (1.44, 2.01) | 1.33* | (1.11, 1.59) | 1.51* | (1.13, 2.00) | 1.18 | (0.99, 1.40) | 1.56** | (1.30, 1.87) |
| **Interaction: COVID*LOS** |  |  |  |  |  |  |  |  |  |  |
| COVID*4-7 days | 1.03 | (0.82, 1.29) | 0.95 | (0.74, 1.22) | 0.73 | (0.46, 1.14) | 0.84 | (0.66, 1.07) | 1.14 | (0.89, 1.47) |
| COVID*>7 days | 0.83 | (0.65, 1.04) | 0.86 | (0.66, 1.11) | 0.66 | (0.43, 1.00) | 0.95 | (0.74, 1.21) | 1.02 | (0.79, 1.32) |
| **Admitted from OPD/transferring (A&E as reference)** | 1.42** | (1.25, 1.61) | 1.26* | (1.10, 1.45) | 1.08 | (0.84, 1.41) | 1.20* | (1.05, 1.37) | 1.41** | (1.23, 1.62) |
| **Interaction: COVID*Admission source** | | |  |  |  |  |  |  |  |  |
| COVID*OPD/transferring | 1.12 | (0.94, 1.33) | 1.06 | (0.88, 1.28) | 1.01 | (0.70, 1.46) | 1.01 | (0.84, 1.21) | 1.03 | (0.85, 1.24) |
| **Self-rated health (Very good as reference)** | | |  |  |  |  |  |  |  |  |
| Good | 0.40** | (0.30, 0.52) | 0.40** | (0.31, 0.53) | 0.55* | (0.31, 0.99) | 0.50** | (0.37, 0.67) | 0.45** | (0.34, 0.60) |
| Fair | 0.25** | (0.19, 0.33) | 0.28** | (0.22, 0.37) | 0.37* | (0.21, 0.65) | 0.38** | (0.29, 0.51) | 0.26** | (0.20, 0.35) |
| Poor | 0.16** | (0.12, 0.21) | 0.18** | (0.13, 0.25) | 0.24** | (0.13, 0.46) | 0.27** | (0.19, 0.38) | 0.12** | (0.09, 0.17) |
| Very poor | 0.13** | (0.08, 0.22) | 0.12** | (0.07, 0.20) | 0.64 | (0.25, 1.62) | 0.24** | (0.14, 0.41) | 0.11** | (0.06, 0.18) |

Note: *P<0.05, **P<0.001; Random effect applied to account for difference across hospitals; AOR: adjusted odds ratio for one unit improvement. The cut-off day in sensitivity analysis is 4 Jan 2021, while 23 Jan 2021 was used in formal analysis.
